# Patient-Reported Quality of Life versus Physical Examination in Treating Temporomandibular Disorders with Intra-Articular Platelet-Rich Plasma Injections: An Open-Label Clinical Trial

**DOI:** 10.3390/ijerph192013299

**Published:** 2022-10-15

**Authors:** Maciej Sikora, Marcin Sielski, Maciej Chęciński, Kamila Chęcińska, Barbara Czerwińska-Niezabitowska, Dariusz Chlubek

**Affiliations:** 1 Department of Maxillofacial Surgery, Hospital of the Ministry of Interior, Wojska Polskiego 51, 25-375 Kielce, Poland; 2 Department of Biochemistry and Medical Chemistry, Pomeranian Medical University, Powstańców Wlkp. 72, 70-111 Szczecin, Poland; 3 Department of Oral Surgery, Preventive Medicine Center, Komorowskiego 12, 30-106 Kraków, Poland; 4 Department of Glass Technology and Amorphous Coatings, Faculty of Materials Science and Ceramics, AGH University of Science and Technology, Mickiewicza 30, 30-059 Cracow, Poland; 5 Specialist Orthodontic Practice: Treatment of Masticatory Organ Dysfunction, Kujawska 19/41, 25-344 Kielce, Poland

**Keywords:** health-related quality of life, temporomandibular joint, temporomandibular disorders, intra-articular injections, platelet-rich plasma, Fonseca questionnaire

## Abstract

Temporomandibular disorders, often manifested by articular pain, limitation of the mouth opening range, and unpleasant acoustic symptoms originating from inside the joint, have been associated with reduced quality of life. These symptoms, among others, can be treated with intra-articular injections of various substances, including repeated platelet-rich plasma (PRP) administration. The reported study was designed as an uncontrolled open-label clinical trial of consecutive cases. The participants completed a Fonseca questionnaire and evaluated acoustic symptoms, and spontaneous and provoked pain on VAS, and were subjected to a physical examination before, during, and after PRP therapy. The total Fonseca questionnaire results were statistically significantly (*p* < 0.05) correlated with 340 out of 348 (98%) other variables. The fully subjective assessment of the presence and intensity of acoustic symptoms coming from the temporomandibular joints was correlated with the physical examination results (0.45–0.63) and the maximum mouth opening with the maximum pain-free mouth opening (0.73–0.87). There were no correlations observed between the patient’s and the physician’s assessment of mandibular mobility. The Fonseca questionnaire seems to cover the entire spectrum of temporomandibular disorders, making it a balanced tool for assessing the quality of life in TMDs. However, it is worth considering extending the standard 3-point response scale to an 11-point one. The patient is somewhat able to perform a self-diagnosis with regard to the acoustic symptoms, but it is not possible for them to determine without measurement whether the range of mandibular mobility is appropriate..

## 1. Introduction

**Figure 1:**
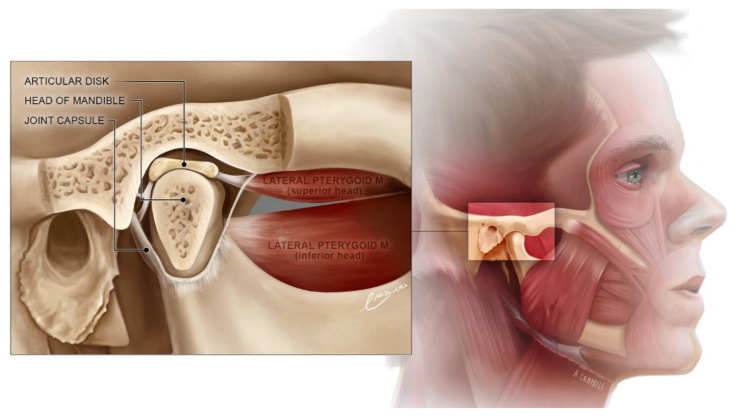
Temporomandibular joint. Cropped. Inset illustration: Emily McDougall, University of Dundee School of Dentistry. Background illustration: Annie Campbell, University of Dundee School of Medicine. CC BY-NC-ND 4.0.

Mandibular movement is a natural, continuously, and often unconsciously performed activity. Cutting, tearing, and grinding the food with ones’ teeth is almost automatic during meal consumption. For the rest of the day, the mandible movements are involuntary in relation to the maxilla. The intensity of night tightening and grinding is associated not only with occlusive but also with emotional conditions [1]. The arrangement of the bones, tendons, ligaments, and muscles required to move the mandible is a complex and precise mechanism (Figure 1). It can compensate for minor disturbances, which are expressed as a change in muscle tone and bone remodeling [2,3]. More significant deviations overwhelm the adaptation possibilities and often result in symptomatic temporomandibular disorders (TMDs). The causes of such an imbalance include tooth loss, poorly adjusted restorations, improperly selected fixed prosthetic elements, excessive muscle tension, depression, bone fractures, and degenerative diseases [1,4,5,6].

The symptoms of temporomandibular disorders (TMDs) are often manifested by articular pain, limitation of the mouth opening range, and unpleasant acoustic symptoms from inside the joint during its activity [1,4,7,8,9]. TMDs have been shown to reduce the quality of life (QoL) of patients suffering from such ailments [5,9,10,11]. However, no standard for health-related QoL assessment in patients with TMDs has yet been developed [5,10]. Among the patient-reported QoL surveys, the Fonseca questionnaire, Oral Health Impact Profile-14, Oral Behaviors Checklist, and numerous general medicine questionnaires are used for the QoL evaluation in TMDs [5,10,11,12,13]. Known studies are static assessments of ailments at a given point in time [5,9,10,12]. However, there are probably no studies that prove the relationship between the results of the questionnaires and the exchange values in the physical examination during therapy. Measuring the relationship between the subjective assessments of a patient and the objective ones of the researcher in a dynamic state of gradual improvement resulting from treatment may be of fundamental importance for further development of the patient-reported QoL surveys in TMDs.

**Figure 2:**
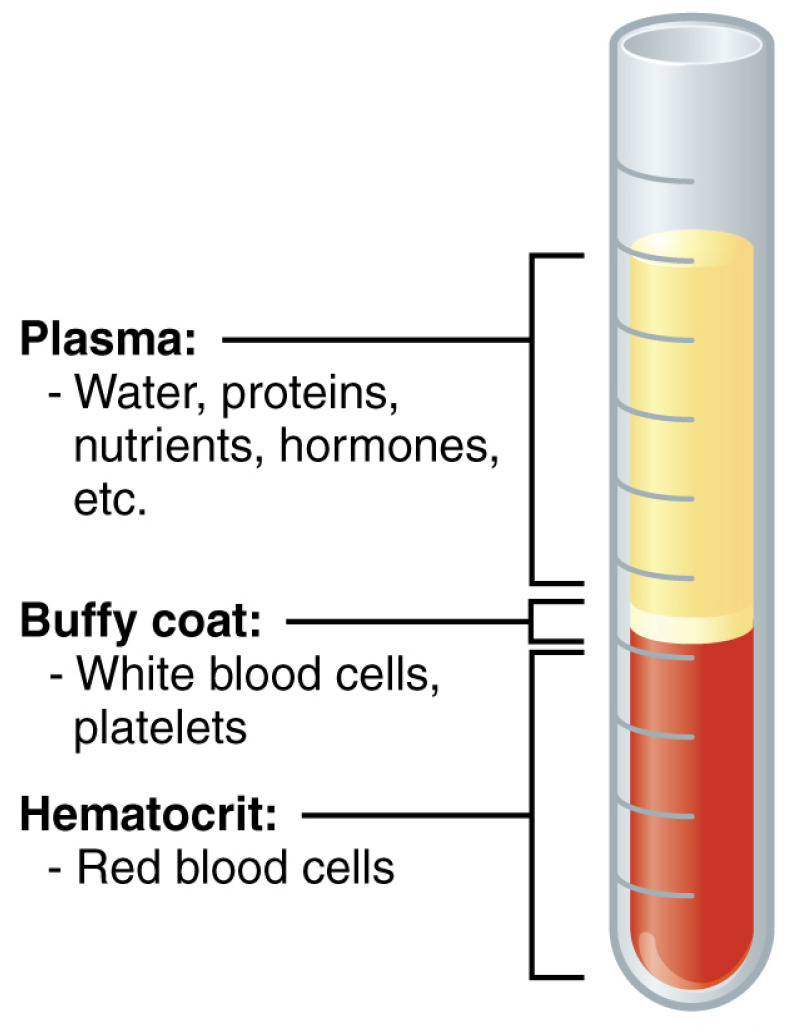
Composition of Blood. Cropped. OpenStax College, CC BY 3.0.

One of the effective therapies in some cases of TMDs are intra-articular injections [14,15,16,17,18,19]. The detailed treatment techniques with proven effectiveness include rinsing the joint cavity, administration of hyaluronic acid, and autologous blood products [7,8,14,17,19,20,21]. Among the latter, platelet-rich plasma (PRP) is the best studied so far (Figure 2) [8,15,16,21,22,23,24,25]. Being a self-derived substance, its use is associated with unlimited availability and a relatively low risk of complications [8,26]. Moreover, PRP is claimed in some studies to be more effective than arthrocentesis and hyaluronic acid administration [25,27,28,29]. The repeatability of interventions in injection therapies allows for the assessment of the dynamics of change in the intensity of subjective complaints and confronting them with the objective results of physical examination [8]. Gradual changes in the intensity of symptoms in PRP therapy present better conditions for observing the improvement in the quality of life than constant dynamic treatment, such as pharmacotherapy or splint therapy [8]. Conversely, repeated administration of other injectables may have a questionable therapeutic benefit as demonstrated by a meta-analysis of hyaluronic acid studies [17]. Thus, repeated administration of PRP seems to be an excellent intervention to observe the differences between the subjective and objective assessment of TMJs’ function.

### Objectives

The study described in this report was conducted to verify the hypothesis that there are differences between the patient’s subjective assessment of symptoms and the results of physical examination in the course of temporomandibular disorders’ treatment by intra-articular administration of platelet-rich plasma.

## 2. Materials and Methods

### 2.1. Study Design and Settings

The study was designed as an uncontrolled open-label clinical trial of consecutive cases. The lack of a control group stems from the intention to perform a parametric comparison of a number of diagnostic methods in a single group of patients. All patients gave their written consent to the treatment and participation in the clinical trial. The report was prepared following the STROBE protocol [30]. The participants completed questionnaires and were subjected to a physical examination before, during, and after PRP therapy. The research was conducted at the Department of Maxillofacial Surgery, Hospital of the Ministry of Interior in Kielce, Poland. The study was carried out in 2020–2022. The entire study program consisted of 6 visits. The interventions took place at visits from 1 to 5. Each patient completed a Fonseca questionnaire at each visit [13]. Additionally, all participants rated the severity of acoustic symptoms and pain in the last 7 days on a visual analog scale (VAS) for both TMJs in the range of 0–10 [31]. At each visit, the range of maximum mouth opening was physically tested. During the initial and final visits, the physician additionally examined the horizontal mobility of the mandible and assessed the presence of acoustic symptoms for each possible direction of movement in each of the TMJs. Additionally, a patient on VAS recorded a current pain value for each of the TMJs immediately after the physical exam. The scope of the questionnaire and physical examination at each visit is presented in Appendix A, Table A1. An interval of 7 to 14 days between visits was planned, depending on the organizational capacity of the facility and the patient’s availability.

### 2.2. Intervention

The vacuum tube with 8 mL of peripheral blood taken from the ulnar vein was centrifuged (160 revolutions for 5 min). After separating the fractions into red blood cells, a buffy coat, and PRP, the latter was aspirated into a syringe. The PRP preparation was administered bilaterally via a single injection through disinfected skin as described below. After the mandible was abducted, a Holmlund–Hellsing line was drawn between the tragus (T) and the lateral corner of the eye [32]. On this line, a point A distant 10 mm from point T was marked. The puncture was made 3 mm below point A, thus reaching the upper TMJ compartment [32,33].

### 2.3. Participants

In a subsequent reporting, adult patients suffering from chronic TMJ disorders were enrolled in the study. In order to obtain a homogeneous study group and enable the necessary analyses, only patients suffering from bilateral TMDs were included in this study. The participants were made aware of the type of therapy they received. The condition for participating in the study was the discontinuation of other TMJ therapies over its course. The detailed inclusion and exclusion criteria are shown in Table 1.

### 2.4. Variables and Data Sources

Gender, age, and history length were classified as descriptive variables. The following were collected as effective subjective assessments and physical examinations: the total score of the Fonseca questionnaire for each visit (FT1–FT5); responses to the Fonseca questionnaire (FQ1-FQ10) on a scale of 0—never, 1—sometimes, 2—often; subjective severity of acoustic symptoms on the visual analog scale (VASA) from 0 (none) to 10 (unbearable) for the right and left TMJ; subjectively perceived intensity of spontaneous pain (VASSP) and pain provoked by physical examination (VASPP) for right and left TMJ on VAS; the range of mandibular mobility in millimeters (mm): maximum mouth opening without pain (MMOwoP), maximum mouth opening (MMO), maximum mandible protrusion (MMP), maximum right-hand movement (MR), maximum left-hand movement (ML); the intensity of acoustic symptoms for opening (AO), protrusion (AP), movement to the right (AR), movement to the left (AL), opening from protrusion (AOP) on a scale of 0—none, 1—mild, 2—strong for both TMJs. Abbreviations for variables are additionally presented in Appendix A, Table A2.

### 2.5. Bias

Two teams of authors worked independently of each other in the study. The first team working with patients consisted of two authors and several assistants from outside the group of authors. Patients were qualified by a specialist in orthodontics (B.C.-N.). The interventions were carried out independently by a specialist surgeon (M.S. (Marcin Sielski)). The other three authors (second team; M.S. (Maciej Sikora), M.C., K.C.) performed data processing and analysis. The report was prepared together by both teams of authors.

### 2.6. Study Size

It was decided to enroll consecutively treated patients, regardless of their number, but with a time limit of 3 months. The condition for inclusion was to begin the therapy on the scheduled date. The dates of the next therapeutic and follow-up visits were irrelevant to the eligibility for synthesis.

### 2.7. Statistical Methods

A correlation matrix with the assessment of their statistical significance was developed. Pearson’s correlation coefficients were calculated and two-tailed unequal sample sizes and unequal variances independent two-sample Student’s *t*-tests were performed. Correlations with the absolute value of the Pearson correlation coefficient greater than 0.5 were considered strong, others weak. The level of statistical significance was adopted as 0.05. Missing data were not supplemented in any way.

## 3. Results

### 3.1. Participants and Descriptive Data

**Figure 3:**
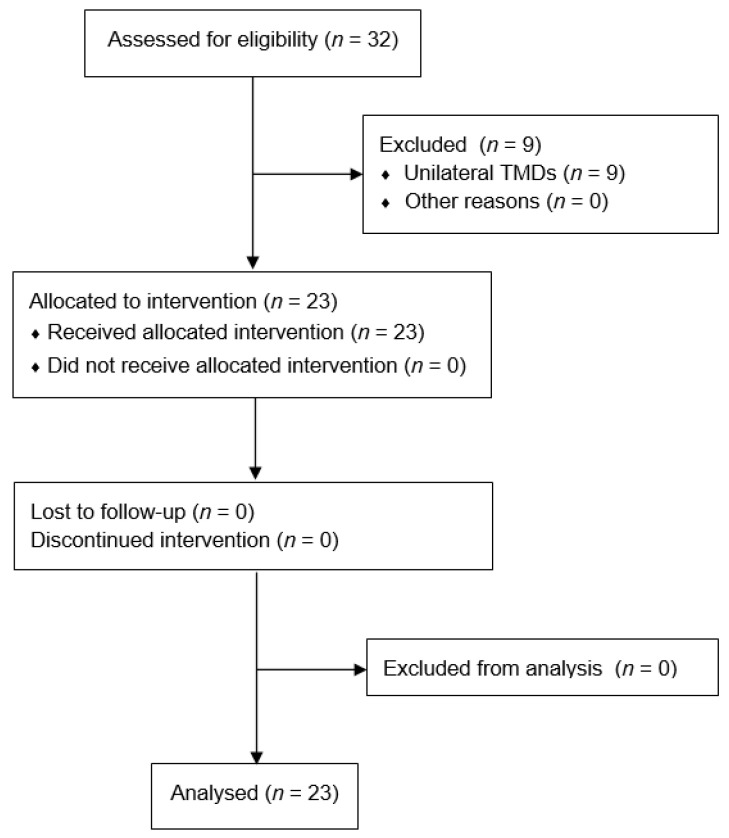
Flow diagram.

Twenty-three Caucasian patients were enrolled in the study, including 20 (87%) women and 3 (13%) men (Figure 3). The age of the participants ranged from 22 to 70 years, the mean age was 40 years, the median was 39 years, and the standard deviation was 13 years. The length of the medical history was from 1 to 30 years, the mean was 8 years, the median was 6 years, and the standard deviation was 7 years. There was no history length for two patients, therefore they were omitted from the calculation for this variable. Pharmacotherapy and splint therapy dominated the previously used methods of TMJ treatment, and they were discontinued during this study. The mean intervals between interventions were 11, 10, 13, and 10 days, medians 11, 10, 12, and 10 days, and standard deviations of 3, 3, 7, and 3 days, respectively. The distinctly different values between the 3rd and 4th intervention resulted from an extension of intervals between visits for two patients, the reasons being non-medical for both. This deviation was not a criterion for exclusion from the study.

### 3.2. Outcome Data

The overall outcome data are shown in Table S1. For the FT1, VASA-R1, VASA-L1, VASSP-R1, VASSP-L1, VASPP-R1, VASPP-L1, MMOwoP1, MMO1, MMP1, MR1, ML1, FT2, VASA-R2, VASA-L2, VASSP-R2, VASSP-L2, MMOwoP2, MMO2, FT3, VASA-R3, VASA-L3, VASSP-L3, MMOwoP3, MMO3, FT4, VASSP-R4, VASSP-L4, MMOwoP4, MMO4, FT5, VASA-R5, VASA-L5, VASSP-R5, VASSP-L5, MMOwoP5, MMO5, FT6, VASA-R6, VASA-L6, VASSP-R6, VASSP-L6, MMOwoP6, MMO6, MMP6, MR6, and ML6 there were no missing data. For the remaining variables, a total of 30 values were missing, which corresponds to 2.08% of all outcome data.

### 3.3. Main Results

**Figure 4:**
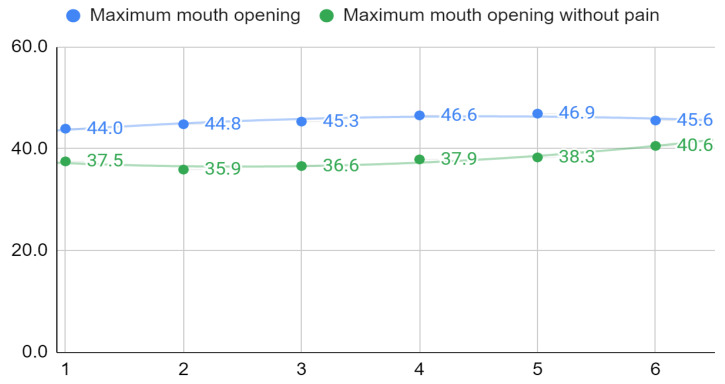
Maximum mouth opening and maximum mouth opening without pain in millimeters on visits 1 to 6.

The complete correlation matrix is provided in Supplementary Materials, Table S2 and *p*-values in Table S3. Values that meet the criterion of statistical significance are shown in bold, and among them, strong correlations are additionally boxed. The total results of the Fonseca questionnaire were statistically significantly correlated with 340 out of 348 (98%) other variables. Almost all of these correlations were weak, and no pattern could be identified among the strong ones. The correlation coefficients between the subjective assessment of acoustic symptoms on VAS and their severity at opening the mouth in the physical examination ranged from 0.45 to 0.63 at different visits and for different parties, and they were all statistically significant. For spontaneous pain and pain provoked by physical examination assessed subjectively on the VAS, no patterns of correlation with other variables were observed. Slightly strong statistically significant negative correlations with the severity of acoustic symptoms in the left joint or with the right-sided movement of the mandible in physical examination appearing at some visits seem to be a coincidence. The maximum mouth opening correlated strongly and statistically significantly with the maximum pain-free mouth opening at each visit (Table 2). The mean values of both variables for the study group are shown in Figure 4. The graph shows the second-degree polynomial trend curves. For MMO and MMOwoP, regression models 42 + 1.9x − 0.2x^2^ (R^2^ = 0.85) and 38.5 − 1.6 + 0.3x^2^ (R^2^ = 0.92) were fitted, respectively. The values of the mobility of the mandible in the transverse plane and the associated acoustic symptoms assessed in the physical examination showed no correlation pattern with any other variables.

### 3.4. Other Analyses

Responses to particular questions of the Fonseca questionnaire (0—never, 1—sometimes, 2—often) were confronted with the results of corresponding elements of the physical examination. Responses to the first question, concerning the difficulty in opening the mouth, were correlated negatively every single time with the maximum mandibular abduction ranging from −0.53 to −0.26. All these correlations were statistically significant. Higher values of answers to this question on a scale of 0 to 2 indicated more frequent difficulty in opening the mouth, and this was correlated with lower abduction values in millimeters. Responses to the second question about the difficulty in performing lateral movements of the mandible showed no correlation with the sum of the values of the right and left slide of the mandible in physical examinations. They were statistically significant correlations of 0.01 and 0.02. The response values to the seventh question regarding the presence of clicking in the joint during opening or chewing strongly correlated with the sum of acoustic symptom scores for abduction for both joints and lateral movements for both sides. These were the correlations of 0.58 for the first visit and 0.63 for the last visit, with the latter failing to be statistically significant. The higher the numerical score on the questionnaire, the higher the number of clicks, and the higher the physician’s score, the greater the severity of clicks assessed. The remaining questions could not be matched with corresponding equivalents in the physical examination.

## 4. Discussion

### 4.1. General Considerations

The endless development of new therapies for increasingly better understood diseases is an indispensable element in the development of medicine. This is also the case for TMDs, which are gradually becoming more and more understandable in terms of classification, etiopathogenesis, and treatment [34,35], and in this field, new therapeutic methods are constantly searched for, including intra-articular injections [6,8,14,17,20,36]. As with any emerging therapy, clinicians face the risk of unexpected adverse events that may result in serious complications [37,38,39,40,41]. A more subtle issue is to assess the effectiveness of emerging treatments and see if they are any better than the existing ones [8,14,16,17,25,42]. The process of therapy validation must be carried out not only objectively and parametrically by researchers, but also subjectively by patients, so as to approach the state of full multidimensional well-being, and not only the absence of disease [5,9,10,11,12]. Only the synthesis of data from the physical examination (including, in particular, the range of mandibular mobility), morphological changes from imaging tests, and the self-assessment of QoL allow for a comprehensive evaluation of the effectiveness of TMDs’ treatment.

The analysis of the quality of life in health and disease and the dynamics of its changes in the course of therapy are an indispensable element of every branch of medicine, including maxillofacial surgery [43,44,45,46,47,48,49,50,51]. There is no shortage of studies on this subject in the fields of traumatology, oncology, and reconstructive surgery [43,44,45,46,47,48,49,50,51]. The tools used in the TMD QoL assessment, discussed in the introduction, seem to be too general [5,10,11,12,13]. The wide scope of the Fonseca questionnaire allows for a holistic approach to QoL in TMDs, but the 3-point scale seems to be insufficient [13].

### 4.2. Key Results

The total Fonseca questionnaire score does not appear to be strongly related to any of the variables included in the physical study [13]. However, it corresponds statistically significantly with almost every one of them. It can therefore be carefully assumed that the design of this questionnaire allows for a balanced assessment of various TMJ disorders and does not favor any of them [13,52,53]. This is consistent with the opinions of other researchers who consider this questionnaire useful at the interview stage [53,54,55]. A detailed assessment of the convergence of answers to three out of ten questions allowed for further-reaching deliberations. Responses to the question with reference to perceptible difficulties in opening the mouth were compared with the absolute values of mandibular abduction, where statistically significant correlations were obtained, ranging from quite weak to average. Considering the discrepancies in the individual values of abduction in healthy people, it can be assumed, based on the obtained results and literature reports, that patients are to some extent aware of the limitation of mouth opening expressed in absolute values.

The absolute values for mandibular abduction also correspond to a semi-subjective assessment of the pain-free mouth opening range. In the conducted study, the latter was a variable assessed in a physical examination, but it largely depended on the patient’s attitude and determination. The results in both domains strongly and statistically significantly correlated with each other at each visit. However, a graphic presentation of the averages for the study group shows differences in the change in MMO and MMOwoP. The first variable changed after each of the first three intra-articular injections to reach the cutoff value on the fourth visit. The next two administrations of PRP did not increase the MMO anymore, and, possibly, the fifth administration even led to a slight deterioration. This can be explained by the longer time interval between the last administration of PRP and the subsequent visit in some patients, which could translate into a gradual disappearance of the effect of this reliever form of treatment. In turn, MMOwoP initially deteriorated, which may be due to the mechanical and inflammatory consequences of the interventions. The values of this variable improved after the third administration of PRP and continued to approach the MMO value. The narrowing of the difference between MMO and MMOwoP should be explained by the relief of joint pain and approaching the full range of pain-free abduction as the therapy result.

The subjective assessment of lateral mandibular mobility, based on the second question in the Fonseca questionnaire, seems to be completely detached from the results of the physical examination. In the discussed study, no correlation between those variables was observed. This issue is difficult to explain, and one should take into account the possibility of high individual variability, a lack of awareness of the correct values of lateral mobility of the mandible, and less pronounced joint ailments compared to those experienced during the abduction. The latter hypothesis assumes that the strongest complaints are related to the movement of the articular disc in the second phase of mandibular abduction.

The presence of acoustic symptoms from TMJs was generally assessed by the patient on the seventh question of the Fonseca questionnaire and the VAS. In both cases, they were quite strong, which proves the consistency of the subjective assessments of the patient and the objective assessments of the physician. The patient assessed only the presence of symptoms and, in the case of VAS, also the side of the body. The physical examination provides additional information about the type of movement that causes the symptom, the stage of movement in which it occurs, and its severity.

Due to having only a 3-point scale for responses to the Fonseca questionnaire, in our study we decided to add questions about the severity of pain and acoustic symptoms with answers expressed on an 11-point scale. Additionally, we duplicated these questions for both TMJs. While the Fonseca questionnaire seems to be an excellent screening tool, several modifications should be considered for a detailed assessment of symptoms and the course of the therapy. In the first question, it is possible to separate the range of mouth opening from the pain, and for the latter variable, to collect the answers for both TMJs. For the second and seventh questions, evaluation for both sides also seems advisable. The answer to each of the questions can be collected in an 11-point (0–10) form, be it VAS or the numeric rating scale (NRS) [56]. Both VAS and NRS are considered reliable in assessing pain and give similar results in comparative studies [57,58].

### 4.3. Limitations

In the study group, no correlation pattern between the gradual relief of joint pain and other variables could be found. This is not consistent with other studies, which indicate a concomitant increase in the range of mandibular mobility in the course of TMJ intra-articular injection therapy [7,8,17]. The lack of such correlations can be explained by the breakdown of both pain and mandibular mobility into many components, which on one hand is the strength of this study, and on the other, its limitation. Other limitations are the small study group resulting from the limitation of the recruitment time and heterogeneous diagnoses.

## 5. Conclusions

The semi-subjective assessment of maximum mouth opening without pain strongly correlates with maximum mouth opening. There were no correlations between the patient’s and the physician’s assessment of mandibular mobility. The fully subjective assessment of the presence and intensity of acoustic symptoms from the temporomandibular joints seems to be quite strongly correlated with the results of the physical examination. In the study group, no patterns of correlation were observed between the patient’s assessment of articular pain and the parameters available from the physical examination. The Fonseca questionnaire seems to cover the entire spectrum of temporomandibular disorders, but it is worth considering adding an extension of the standard 3-point response scale to an 11-point scale.

## Figures and Tables

**Table 1:** Inclusion and exclusion criteria.

Inclusion Criteria	Exclusion Criteria
- consent to participate in the study - 18 years of age or older - diagnosis of bilateral osteoarthritis, internal derangements, or degenerative joint disease - declaration of discontinuation of other forms of treatment of TMJ diseases for the duration of the study - no changes in the treatment of other chronic diseases expected during the study period	- resignation from participation in the study - ailments that have been present for less than a year - blood diseases, local diseases in the preauricular area - deviation from any of the inclusion criteria during the study, in particular, changes in the treatment of any chronic disease

**Table 2:** Correlations of maximum mouth opening (MMO) with maximum mouth opening without pain (MMOwoP).

	MMOwoP1	MMOwoP2	MMOwoP3	MMOwoP4	MMOwoP5	MMOwoP6
MMO1	0.74					
MMO2	0.59	0.73				
MMO3	0.63	0.59	0.82			
MMO4	0.63	0.35 *	0.55	0.81		
MMO5	0.59	0.59	0.68	0.65	0.82	
MMO6	0.51	0.45 *	0.57	0.66	0.57	0.87

The numbers next to the abbreviations indicate the visit number. Correlations that are not statistically significant are marked with an asterisk. The correlations on gray backgrounds constitute an important correlation pattern and are discussed in the text.

## Data Availability

All data can be found in the Supplementary Materials. The quantitative data collected in the study are presented in Table S1. The results of the static analyses are presented in Table S2 (the statistically significant correlation values are in bold, and those classified as strong are outlined).

## Supplementary Materials

The following supporting information can be downloaded at: https://www.mdpi.com/article/10.3390/ijerph192013299/s1. Table S1. Outcome data; Table S2. Correlation matrix; Table S3. *p*-Values.

## Appendix A

**Table A1:** The scope of the questionnaire and physical examination at medical visits. TMJ—temporomandibular joint; VAS—visual analog scale.

Visit	Questionnaire	Physical Examination	Intervention
1	Fonseca questionnaire Right and left TMJ acoustic symptoms on VAS Right and left TMJ spontaneous and provoked pain on VAS	Maximum mouth opening without pain Maximum mouth opening Maximum protrusive, right, and left mandibular movements Acoustic symptoms testing	Yes
2	Fonseca questionnaire Right and left TMJ acoustic symptoms on VAS Right and left TMJ spontaneous pain on VAS	Maximum mouth opening without pain Maximum mouth opening	Yes
3	Fonseca questionnaire Right and left TMJ acoustic symptoms on VAS Right and left TMJ spontaneous pain on VAS	Maximum mouth opening without pain Maximum mouth opening	Yes
4	Fonseca questionnaire Right and left TMJ acoustic symptoms on VAS Right and left TMJ spontaneous pain on VAS	Maximum mouth opening without pain Maximum mouth opening	Yes
5	Fonseca questionnaire Right and left TMJ acoustic symptoms on VAS Right and left TMJ spontaneous pain on VAS	Maximum mouth opening without pain Maximum mouth opening	Yes
6	Fonseca questionnaire Right and left TMJ acoustic symptoms on VAS Right and left TMJ spontaneous and provoked pain on VAS	Maximum mouth opening without pain Maximum mouth opening Maximum protrusive, right, and left mandibular movements Acoustic symptoms testing	No

**Table A2:** Variables with abbreviations and possible values. (P)—Patient assessment; (E)—Physical examination; R—right side; L—left side.

Variable	Abbreviation	Possible Values
Fonseca questionnaire total score (P)	FT1-FT-5	0 (no symptoms)–20 (strong symptoms)
Visual Analog Scale Acoustic symptoms (P)	VASA-R, VASA-L	0 (none)–10 (unbearable)
Visual Analog Scale Spontaneous Pain (P)	VASSP-R, VASSP-L	0 (none)–10 (unbearable)
Visual Analog Scale Provoked Pain (P)	VASPP-R, VASPP-L	0 (none)–10 (unbearable)
Maximum Mouth Opening without Pain (P,E)	MMOwoP	millimeters
Maximum Mouth Opening (E)	MMO	millimeters
Maximum Mandible Protrusion (E)	MMP	millimeters
Maximum Right-hand movement (E)	MR	millimeters
Maximum Light-hand movement (E)	ML	millimeters
Acoustic Symptoms for Opening (E)	AO-R, AO-L	0—none, 1—mild, 2—strong
Acoustic Symptoms for Protrusion (E)	AP-R, AP-L	0—none, 1—mild, 2—strong
Acoustic Symptoms for Right-hand movement (E)	AR-R, AR-L	0—none, 1—mild, 2—strong
Acoustic Symptoms for Light-hand movement (E)	AL-R, AR-L	0—none, 1—mild, 2—strong
Acoustic Symptoms for Opening from Protrusion (E)	AOP-R, AOP-L	0—none, 1—mild, 2—strong
